# Experimental Investigation of Impactor Diameter Effect on Low-Velocity Impact Response of CFRP Laminates in a Drop-Weight Impact Event

**DOI:** 10.3390/ma13184131

**Published:** 2020-09-17

**Authors:** Hongyi Cao, Mengyuan Ma, Mingshun Jiang, Lin Sun, Lei Zhang, Lei Jia, Aiqin Tian, Jianying Liang

**Affiliations:** 1 School of Control Science and Engineering, Shandong University, Jinan 250061, China; caohongyi@mail.sdu.edu.cn (H.C.); mamengyuan@mail.sdu.edu.cn (M.M.); drleizhang@sdu.edu.cn (L.Z.); jialei@sdu.edu.cn (L.J.); 2 Zhongche Qingdao Sifang Locomotive and Rolling Stock Co., Ltd., Qingdao 266111, China; sfsunlin@aliyun.com (L.S.); sftianaiqin@sina.com (A.T.); sfliangjianying@sina.com (J.L.)

**Keywords:** composites, low-velocity impact, impact response, non-destructive testing, ultrasonic

## Abstract

The present study delved into the effect of impactor diameter on low velocity impact response and damage characteristics of CFRP. Moreover, the phased array ultrasonic technique (PAUT) was adopted to identify the impact damages based on double-sided scanning. Low-velocity impact tests were carried out using three hemispherical impactors with different diameters. The relationship of impact response and impactor diameters was analyzed by ultrasonic C-scans and S-scans, combined with impact response parameters. Subsequently, the damage characteristics were assessed in terms of dent depth, delamination area and extension shape via the thickness, and the relationships between absorbed energy, impactor displacement, dent depth and delamination area were elucidated. As revealed from experiment results, double-sided PAUT is capable of representing the internal damage characteristics more accurately. Moreover, the impactor diameter slightly affects the impact response under small impact energy, whereas it significantly affects the impact response under large impact energy.

## 1. Introduction

Carbon fiber reinforced polymer (CFRP) has been extensively employed in lightweight design (e.g., aerospace, automotive and rail traffic) for its merits of high specific strength and stiffness. However, CFRP is susceptible to damage caused by out-of-plane impact force, which is one of the major concerns in the design of structures made of CFRPs [1,2]. Its vulnerability to low velocity impact events will induce Barely Visible Impact Damages (BVID) in the composite structures, which is manifested mostly by delamination and matrix crack with limited fiber breakage [3,4,5] that cannot easily be detected macroscopically. BVIDs pose significant safety issues since they are capable of generating extended damage and decreasing the residual strength and durability of the structure, which may cause catastrophic failure [6,7,8].

Damage response behavior of composites under impact remains unclear for the multitude of CFRPs that can be synthesized by a variety of stacking sequence and manufacturing procedures. Impact damages of CFRP refer to a sophisticated mechanism that consists of matrix cracking, delamination and fiber breakage. Moreover, many other factors (e.g., impact parameters, boundary conditions) will affect the damage characteristics [9]. Nondestructive testing (NDT) is considered a critical testing method to detect and characterize internal damages in CFRPs, which has been employed in different stages of the life of a composite structure. First, NDT techniques are applied in combination with impact/fatigue tests to estimate its performance under load; then, they are used before assembly to assure that the material is free from manufacturing defects; subsequently, they are adopted to detect in service deterioration [9,10].

To monitor the damage process and characterize the BVIDs, several NDT techniques such as ultrasonic testing [11,12,13], infrared thermography [10,14], Lamb waves [15], acoustic emission [16], have been studied extensively. In fact, each technique exhibits unique advantages and inherent limitations, which is determined by their testing principles and applications [17,18]. Ultrasonic testing refers to a significantly applicable and effective technique, which is also extensively employed in practical defect inspection. Phased array ultrasonic system has been developed rapidly with the rapid growth in the microelectronics and computer technology. For multi-element signal focusing, phased array ultrasonic testing exhibits more favorable attenuation and resolution characteristics of ultrasonic signals compared with conventional ultrasonic [9]. Over the past few years, several studies have discussed the application of phased array ultrasonic technique to composite structures. Meola et al. [10] studied the merits of using phased array ultrasonic technique compared with thermography; they also determined that an integrated analysis of C-scan, S-scan helps assess the overall view of the internal damages and their location via the thickness. Caminero et al. [9] analyzed the ability of PAUT to recognize and assess non-visible artificial inclusions and damages in composite laminates; they drew the conclusion that the use of PAUT to assess internal damage is appropriate and advantageous, which can explain the complexity of impact events in different composites. Sadeghi et al. [19] proposed a double-side methodology to perform reliable damage/depth detection on CFRP plates under low-velocity impact with PAUT; they suggested that the damage dimensions grow both horizontally and vertically with increasing impact energy. The mentioned studies primarily discussed the detection of damage area, however, the extension shape of internal damage was rarely investigated carefully.

Impactor parameters (e.g., shape, diameter and mass) are capable of affecting the impact responses of laminates significantly [1,2]. Mitrevski et al. [20] delved into the effect of impactor shape on the damage resistance and tolerance of woven carbon/epoxy laminates; they experimentally verified that the absorbed energy, peak force and damage threshold load of all are susceptible to the impactor shape. Furthermore, a post-impact analysis of the mentioned laminates was conducted with NDT and destructive inspection [21], and it was reported that different impactor shapes generated different levels of the individual damage components, thereby affecting the residual properties of the laminates. Sławski et al. [22,23] presented some useful results obtained by experimental and numerical research focusing on the influence of the strikers’ geometry; on the basis of research using an optical microscope, they found that the strikers’ geometry has a significant effect on the damaged microscopic images, and the damage caused by the strikers with a pointed end was much greater. Icten et al. [24] investigated the effect of impactor diameter on the impact response of woven glass-epoxy laminates; they investigated variation of the impact characteristics and CAI after impact test, and concluded that the impactor diameter highly affects the impact and CAI response of composites materials. Zhou et al. [25] studied the effect of four factors including impactor diameter on the impact responses of CFRP laminates, and their experimental results demonstrate that the four factors have significant effects on the impact behaviors of laminates in different ways; however, the effect of impactor diameter at different impact energies was not involved. The above-mentioned researches on the impactor diameter mostly focus on the impact response and compressive strength after impact, and there is a lack of detailed research on the effect of the shape and expansion of internal damage, which will have an important impact on the residual strength of composite structures. Several studies also explored the effect of impact mass and velocity on the low velocity impact of CFRPs [25,26,27], drawing similar conclusions that the mentioned impactor parameters significantly affect the impact results.

Quasi-static indentation [28] and drop-weight impact [29] are two commonly used method to inflict damage into a specimen. Both of them are widely used to measure damage resistance properties or residual strength properties in recent years [19,25,30,31,32]. In the present study, drop-weight impact tests were performed to induce BVIDs into the composite specimens, and phased array ultrasonic testing was adopted to inspect the specimens subjected to low velocity impact. With a combination of ultrasonic C-scan and S-scan images obtained from front and back side scanning, impact damage was qualitatively and quantitatively assessed. Furthermore, to analyze the effect of impactor diameter on CFRPs, three different impactor diameters were adopted to execute the impact event under three different impact energies for each diameter. To comprehensively and effectively assess the damage status, different damage evaluation criteria (e.g., absorbed energy, final displacement and dent depth) were discussed according to the PAUT results.

## 2. Materials and Methods

### 2.1. Specimen Preparation

**Figure 1:**
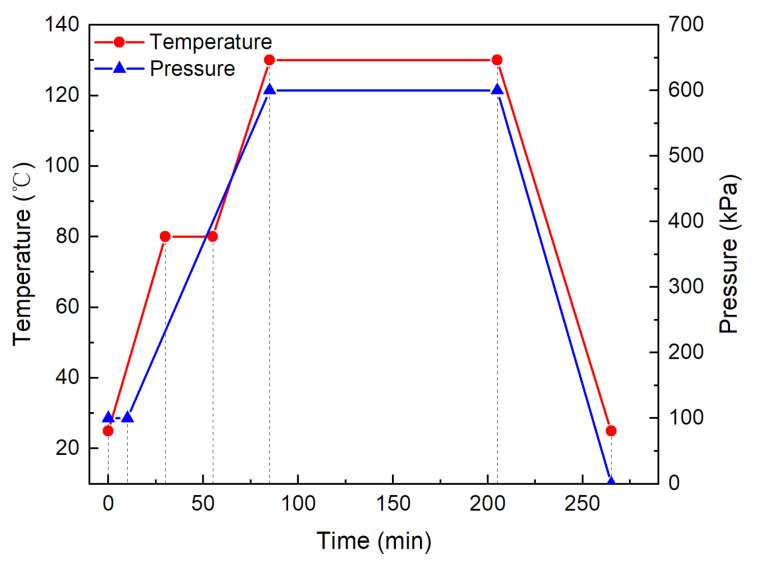
Curing cycle settings of autoclave.

The specimens employed in the present study were made of unidirectional carbon/epoxy prepreg laminas T300/YH69 by autoclave molding. The prepreg tapes exhibit a nominal cured ply thickness of 0.14 mm with a density of 1678 kg/m^3^. The basic in-plane stiffness and strength of the T300/YH69 prepreg are listed in Table 1. As recommended in ASTM D7136 [29], composite laminates were manufactured by stacking 32 plies following a stacking sequence of [45/0/−45/90]${}_{4S}$, with lay-up direction definition that the 0° fiber orientation is aligned with the lengthwise dimension. The specimens were fabricated with the hand layup method and curing in autoclave. The curing cycle settings are presented in Figure 1. Both curing temperature and pressure were peaked at 85 min, and maintained for 2 h at 130 °C and 600 kPa.

A cutting machine with a 3mm diameter corn milling cutter was used to cut the specimens into 150 × 100 mm size. In order to avoid notches, rough or uneven surfaces and delamination due to manufacturing process and inappropriate machining methods, all of the specimens were inspected by the phased array ultrasonic technique and visual inspection before the impact tests, and only specimens without defects would be chosen. The thickness of each plate was determined with a caliper in the middle of all four edges, and the average value obtained was approximately 4.48 ± 0.03 mm.

### 2.2. Low-Velocity Impact Testing

**Figure 2:**
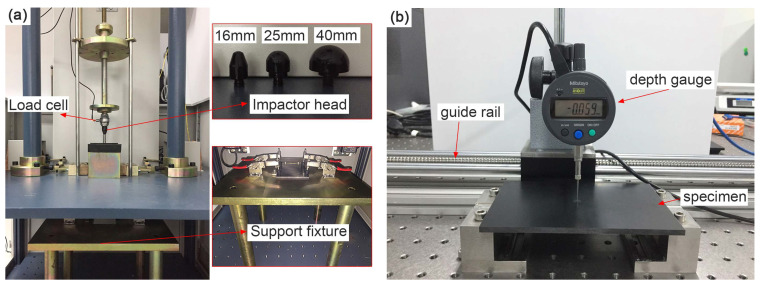
Experiment set-up: (**a**) Low-velocity impact setup, (**b**) instrument for measuring dent depth.

The low-velocity impact event was performed according to ASTM D7136 with a drop-weight testing machine ZBG-0390, which was equipped with a 30 kN load cell to register the contact force history, as well as an anti-rebound device to avoid multiple collisions (Figure 2a).The impact force data was acquired using a 16-bit analog-to-digital converter, NI 9223, and the sampling rate was set to be 100 kHz. Specimens were simply supported on the support fixture with a cut-out of 125 × 75 mm, and four clamps were adopted to restrain the specimen. The total mass of the impactor consists of impactor head, load cell, connecting rod and weights. Three hemispherical impactor heads exhibiting the diameter of 16 mm, 25 mm and 40 mm were used, and three impact energies of 7 J, 17 J and 27 J were taken for each diameter. To avoid the effect of impact velocity [26,27], a constant mass of 2.2 kg was selected for all the impact tests, and the striker was released from different heights to reach different impact energies. The recorded force time history was converted into impact parameters (e.g., displacement and absorbed energy) in accordance with Newton’s second law; the striker was assumed as a free falling rigid body [26].

Three specimens were tested for respective impact condition; then, a total number of 27 specimens were impacted. All the specimens were inspected with phased array ultrasonic before impact testing to avoid manufacturing defects. After impact event, the dent depth of each specimen was determined in 10 min with the high-precision micrometer gauge Mitutoyo 543-791 (Figure 2b). The gauge was fixed on an electric guide rail driven by a stepper motor with a step accuracy of 0.01 mm, with which the depth of the cut surface of the impact dent could be determined.

### 2.3. Phased Array Ultrasonic Testing

Phased array ultrasonic testing was performed with an Olympus OmniScan MX2 portable ultrasonic device. A phased array system refers to a multi-channel ultrasonic system, abiding by the principle of time-delayed triggering of the transmitting transducer elements, combined with a time corrected receiving of the detected signals. The promising aspect of the PAUT indicates its ability to generate the ultrasonic beam where parameters are controlled electronically (e.g., angle of incidence and focal distance). Accordingly, the ultrasonic beam can be focused or steered across the material to be inspected and meet the possibility of testing complex shaped parts and structures. For the mentioned unique features, phased array technique exhibits high productivity, reliability and high resolution compared with conventional ultrasonic NDT methods [33].

**Figure 3:**
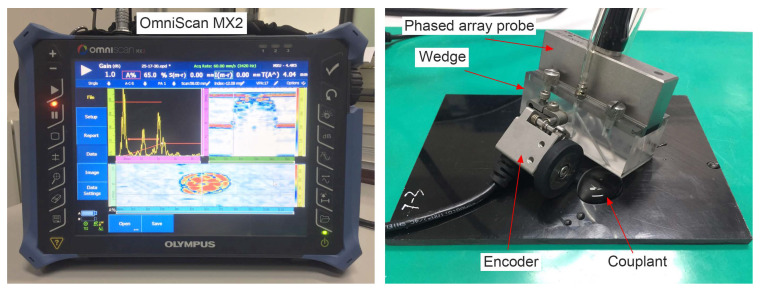
Phased array ultrasonic system.

For composite materials, an applicable useful frequency range of ultrasonic waves is from 1 MHz to 10 MHz [12]. In the present study, phased array probe 5L64-NW1 at a center frequency of 5 MHz was exploited, consisting of 64 elements arranged in a line array and equipped with a maximum active aperture of 64 × 7 mm^2^. The complete phased array ultrasonic testing system is illustrated in Figure 3. The probe was connected to a wedge to detect near-surface defects via pulse delay. To couple the sound into the specimen, purified water acted as coupling medium. The position of the probe on the plates was identified by a wheel encoder (Olympus ENC1-2.5-DE) connected with the wedge and the probe. Before the inspection process, the ultrasonic propagation velocity in the specimen was calibrated to 2960.5 m/s.

**Figure 4:**
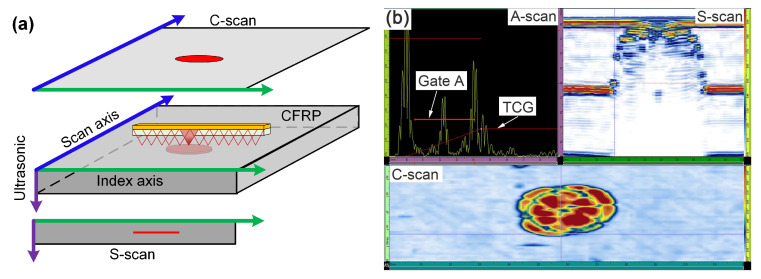
Ultrasonic views: (**a**) Diagram of ultrasonic views, (**b**) A-, S- and C-scan in ultrasonic testing.

On the whole, a reflection echo was attributed to acoustic impedance variations of the material. Ultrasonic views, as shown in Figure 4, are images defined by different plane views between the ultrasonic path and scanning parameters [33]. A-scan refers to a curve of the received echo amplitude versus time or ultrasonic path, which is termed as a waveform. C-scan and S-scan are two-dimensional graphics calculated from ultrasonic data, respectively representing top view and end view of the test specimen. Thus, C-scans are the post suitable image to determine the shape, the size and the extent of the internal damage, and allow an evaluation of the damaged area parallel to the plate surface, while S-scans can generate a cross-section view of the specimen that can show the extent of the internal damage at the depth direction.

In this study, it was configured in a way that the sound beam was pulsed by a group of 8 active elements electronically from the 1st to the 64th element. Accordingly, a length of about 57 mm cross-section scans can be without moving the probe. The damage is assumed to be in the middle of the plate with a diameter less than 57 mm. In this scenario, single line scan in the middle of the plate will be sufficient to assess the damaged area in all the other plates. The direction of scan axis is aligned on the lengthwise dimension. Time Controlled Gain (TCG) technical was adopted to compensate the high sound attenuation attributed to the multi-ply of the composite laminate and the larger distance between the sound emitting source and reflector. The gain settings of the ultrasonic device fitted the travelling time of the sound, and their correlations were identified. After applying TCG, the reflect echo of the bottom side is visualized with the identical intensity on the upper side.

## 3. Results and Discussion

### 3.1. Damage Response Parameters

#### 3.1.1. Impact Force-Time History

**Figure 5:**
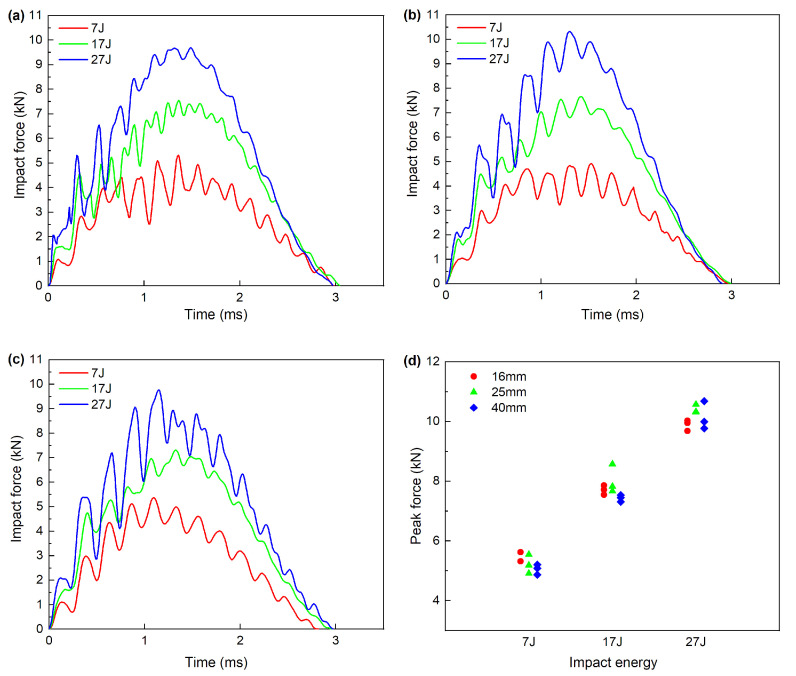
Force history under different impactor diameters: (**a**) 16 mm, (**b**) 25 mm, (**c**) 40 mm, (**d**) peak force.

The typical impact force-time cures during impact event are shown in Figure 5a-c, in which only one of the three specimens under the same impact condition was shown. According to the figures, the contact force at the beginning of the curve increases up to the maximum value with intense oscillations. The oscillations indicate that some internal damages (e.g., cracking and delamination) have occurred. The experimental results show that internal damages have been induced for all the impacted specimens. Several differences can be observed from the impact force responses of these specimens, probably impacted by their damage mechanisms. After reaching the peak force, the curve oscillation of diameter 40 mm is obviously different from that of diameter 16 mm and 25 mm at impact energy 27 J. In Figure 5c, the oscillation remains shortly at impact energy 27 J, probably indicating more serious internal damages. The cures contain numerous oscillations at a lower frequency, which may be introduced by the flexural vibration of the impacted specimen.

The contact duration of all the impact events is approximately 3 ms, and no considerable difference is identified between different impact conditions. Figure 5d presents the peak force for different diameters under three impact energies. It is observed that as the impact energy increases from 7 J to 27 J, the peak force increases linearly. For the same impact energy, the peak force of different impactor diameters exhibits slight difference under the mentioned impact conditions. However, from the analytical theory of out of plane indentation [34,35], the peak force will increase with a larger impactor diameter because the contact stiffness increases at the similar energy level. The difference between the analytical theory and the experimental result may cause by the different boundary conditions. In the present work, the specimens were simply supported and fixed, which may affect the contact stiffness and bending stiffness during the impact event.

#### 3.1.2. Impact Force-Displacement Response

**Figure 6:**
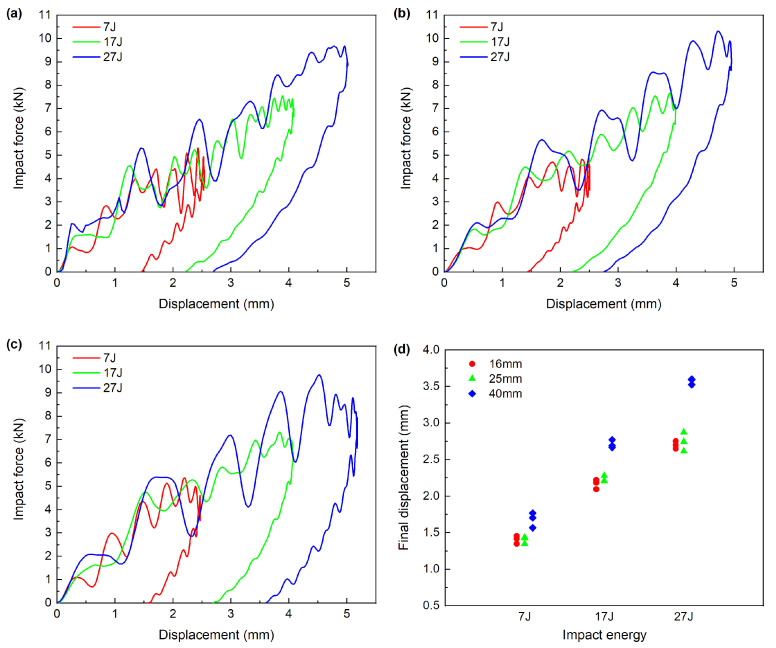
Force-displacement curves under different impactor diameters: (**a**) 16 mm, (**b**) 25 mm, (**c**) 40 mm, (**d**) final displacement.

The central displacement of the impactor was calculated by the formula given in the ASTM D7136. The typical force-displacement curves during the impact are demonstrated in Figure 6a–c, which focuses on different impactor diameter respectively. The curves can generally be divided into three interval according to the damage initiation, damage evolution and specimen rebounding. In the first stage, the specimen undergoes elastic deformation until the impact force reaches the damage initiation threshold. In the second stage, the damage initiates and evolves inside the specimen, i.e., damage propagations. The vibration on the curve is related to the matrix cracking, delamination and fiber fracture inside the specimen. In the final stage, the specimen begins to rebound, and the elastic energy stored in the specimen returns until it rebounds completely. The corresponding displacement at the end of the curves is defined as the final displacement, where the impact force goes back to zero.

For all impactor diameters, with the rise in the impact energy, the maximum and final displacement both increase. Under different impactor diameters, the maximum displacement and the time to reach it are basically the same under low impact energy. At high impact energy (27 J), however, large diameter (40 mm) will represent larger maximum displacement and longer arrival time. The maximum displacement of impact by 40 mm impactor is 5.286 mm, which is about 4.88% larger than that of the other two impactor. Larger maximum displacement will cause high bending stress at the back of specimen, which may induce larger delamination in the specimen. This conclusion is consistent with the results of ultrasound C scan in Section 3.3.

The linear relationship between the final displacement and the impact energy can be identified for all impactor diameters in Figure 6d. However, the slop of the diameter of 40 mm is higher. The final displacement of diameter 40 mm under the identical energy level is slightly higher, while the other two diameters are nearly identical.

#### 3.1.3. Absorbed Energy

**Figure 7:**
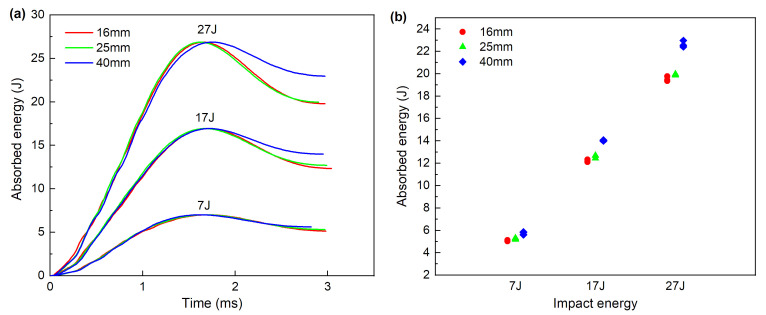
Absorbed energy: (**a**) Absorbed energy time curves, (**b**) final absorbed energy.

Typical absorbed energy curves under the impact are plotted in Figure 7a, as calculated abiding by the ASTM D7136. Once the contact between the impactor and the composite specimen takes place in the impact event, the kinetic energy of the impactor is transferred to the composite plate. The transferred energy is partially absorbed by the composite laminate, thereby causing the internal damage. The other part of the energy is stored as elastic energy; thus, rebounding phenomena are generated [25]. The final energy value denotes the overall amount of energy dissipated by specimens largely by damage evolution. The impactor is assumed to be in free fall without friction and other resistance factors, so the maximum absorbed energy in the curves is identical to the initial impact energy. The consumed time to reach the maximum value for different impactor diameters is considered the same under equienergetic impact, except for the specimen impacted with diameter 40 mm at an energy of 27 J, which takes a little longer time (0.11 ms) than the other two.

Figure 7b presents the final absorbed energy as a function of the impact energy for the different impactor diameters considered. Figure 7b suggests that with the increase in impact energy, the final absorbed energy of all impactor diameters increases linearly. Under equienergetic impact, the final absorbed energy increases slightly with the rise in impactor diameters. As impacted by the difference in the contact area between the impactor and laminates, the impactor diameter will lead to different intralaminar damages, and subsequently affect the final absorbed energy. The results of the subsequent ultrasonic evaluation are consistent with those analyzed.

### 3.2. Impact Damage at Surface

**Figure 8:**
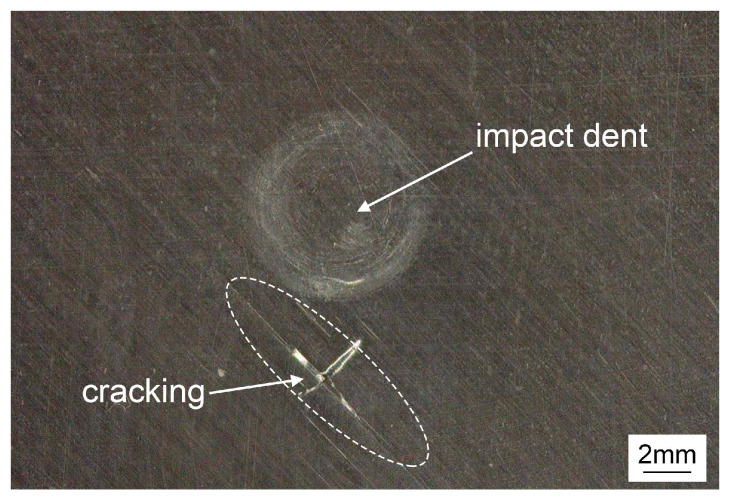
Front surface damage of the 27 J impact with the 16 mm impactor.

Impact damages can be observed both at the front and back surfaces. The damage result of the 27 J impact with the 16 mm impactor at the front surface shows some difference with the other specimens, in addition to permanent indentation, some cracks occurred along the direction of the fiber layer, as shown in Figure 8. The damage can also be observed in the ultrasonic C-scan image from front surface in Section 3.3. The visual impact damages for other specimens at the front surface are only impact dents exhibiting different sizes. The differences of the front surface damages are chiefly caused by the different contact stiffness under different impact conditions.

**Figure 9:**
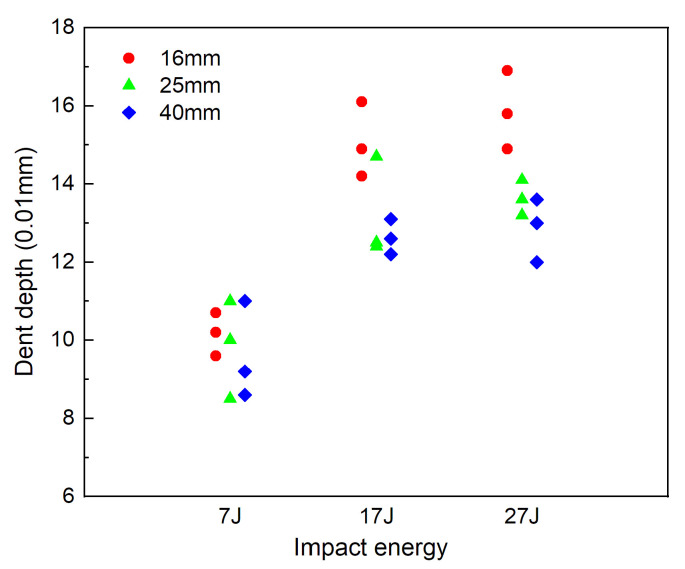
Depth of dents at front surface.

The dent depth determined by the micrometer gauge is illustrated in Figure 9. Dent depth increases with the increase in impact energy, whereas, the increase is decelerated under large impact energy. On equienergetic impact loads, the dent depth decreases with the increase in the impactor diameter. This is primarily because larger diameter impactor and the specimen exhibit broader contact area and lower locally stresses near the impact point in the specimens.

**Figure 10:**
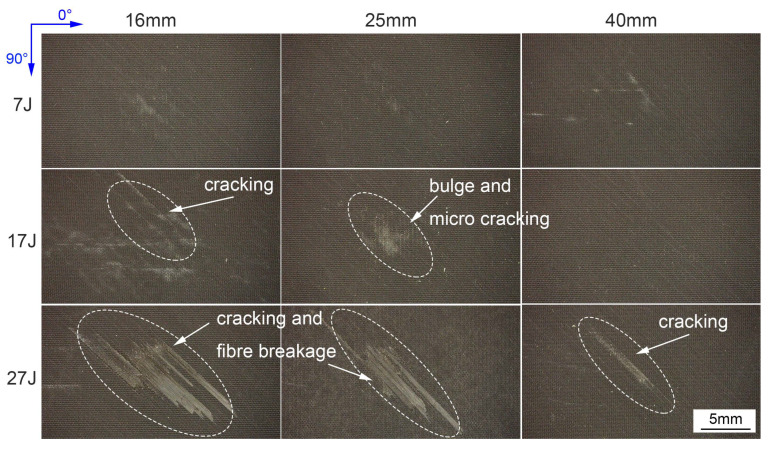
Impact damages at back surface.

Figure 10 presents some typical damages at back surface under different impact conditions. Unlike the front surface damage, damages at backside are considered more serious and exhibit different forms. According to the photos, under all impactor diameters, only some micro cracks and bulges can be observed at lower impact energy. As impact energy increases, some serious cracking and fiber breakage occur, especially with small impactor diameter. The direction of all the cracks is 45°, which is consistent with the layer direction of the last (32th) lamina. Given the front and back surface damages, it is summarized that the impactor diameter noticeably affects the impact failure, small impactor diameter can induce serious surface damage for high local stresses and indentation effects, while larger impactor diameter will induce slight visual damages at the surface.

### 3.3. Ultrasonic Inspection Results

#### 3.3.1. Ultrasonic C-Scans

Ultrasonic C-scan image is capable of presenting the shape, size and transverse expansion of the internal damage. There are two different views of C-scan image, i.e., amplitude signal and depth signal; besides, alterations in the register values are represented by different colors for both of them. Only the maximum amplitude for each point is projected on this scan-index plan view. Compared with amplitude image, depth image can provide more information of the internal damage. Under the amplitude of a reflected echo in Gate A greater than the threshold value, the depth was calculated by considering the flight time and the propagation velocity, and then it was coded by corresponding colors. Thus, the set value of Gate A will exert a direct effect on the result of the damage size.

**Figure 11:**
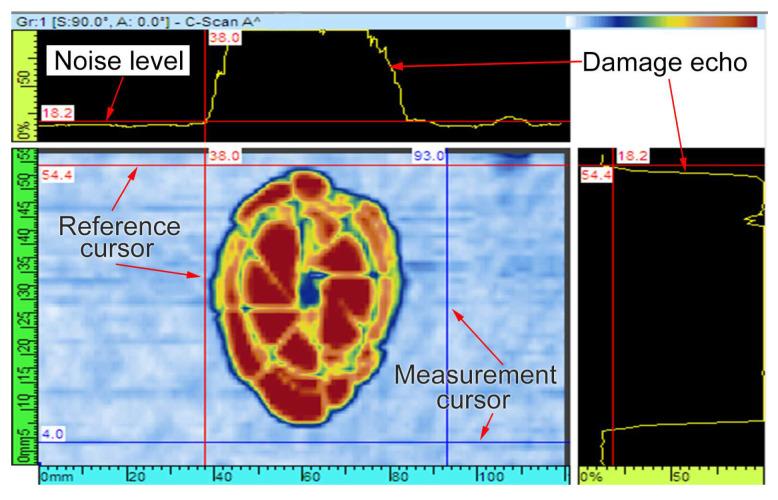
Echo dynamic of the ultrasonic echo.

In the present study, professional processing software (TomoViewer 2.10) developed by Olympus was adopted to delve into the original ultrasonic data. To analyze the damage area of the impacted specimen accurately, echo dynamic was adopted to determine the threshold value of Gate A (Figure 11). The echo-dynamic curves exhibit the maximum amplitude between the reference and measurement cursors [36]. The noise amplitude can be read from the echo-dynamic curves, and the threshold value of Gate A will be set to be slightly larger than the noise around the impact damage.

**Figure 12:**
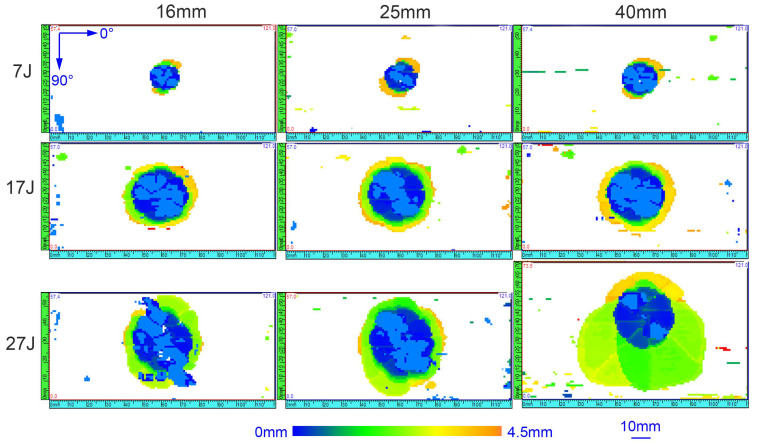
C-scan images from front surface.

In Figure 12, the depth C-scans of representative impacted specimens are visualized. According to the figure, the overall damage area grows significantly with the increase in the impact energy. The color of the image from blue to yellow represents delamination of at different depths from near-surface to near-back. The damage area at different plies increases with the increase in depth via the thickness, so a frustum-cone shape pattern can be identified. Such extension shape of internal damage is elucidated with the cross-section scans in the following.

However, the specimen impacted with diameter 40 mm and energy 27 J suggests some differences, in which the length of the damage area in index direction reaches over that of the active aperture (57 mm). Therefore, two single line scans for the specimen was performed and then merge the acquired C-scan data into one file. The delamination is suggested to spread more severely along 45° direction. The damage shape and expansion suggest slight difference between different impactor diameters for other specimens.

Some isolated pixels can be observed in Figure 12, isolated from each other. The mentioned pixels are attributed to the insufficient coupling conditions, and some are fake internal defects for the very small threshold value of Gate A. Thus, they were not considered in the calculation of the damage area. In this study, rectangular frames around the damaged areas was considered and calculated the damage size based on the areas between the frames.

**Figure 13:**
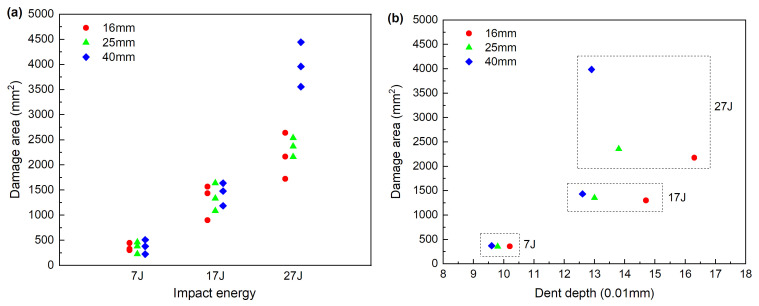
(**a**) Damage area of all specimens, (**b**) Typical correlations of damage area vs. dent depth.

The calculated damage area of all specimens is presented in Figure 13a. For all impactor diameters, damage area increases with the rise in the impact energy. At lower impact energies, the damage areas for all the three impactor diameters are appromately identical; however, when impacted with large energy, larger impactor diameter can induce significantly larger internal delamination than small ones, which complies with the previous analysis of larger displacement and final absorbed energy. On the whole, two different stresses can induce the delamination in the impact event. One is interlaminar shear stress, while the other is bending stress. The bending stress will accelerate the initiation and evolution of damage at the interface between plies, causing a significantly larger delamination in the laminate.

The delamination area depending on the permanent indentation is illustrated in Figure 13b. Under different impact energy, the correlation of damage area and dent depth is very different with different impactor. At impact energy 7 J and 17 J, the damage areas under different impactors are almost the same, while the dent depth decrease with the increase of the impactor diameter. When the impact energy increase to 27 J, the damage area is negatively correlated with the dent depth. This is mainly because there are two main energy dissipation ways in the impact process, i.e., the intralaminar damage evolution (including matrix cracking, fiber breakage and fiber-matrix debonding) and interlaminar damage evolution (delamination). The permanent indentation mainly reflects the intralaminar damage state, and delamination area mainly reflects the interlaminar damage state [25]. Impactor diameter has different impact on contact stiffness, shear stiffness and bending stiffness under different energies, which will induce different intralaminar and interlaminar damage state.

#### 3.3.2. Ultrasonic S-Scans

**Figure 14:**
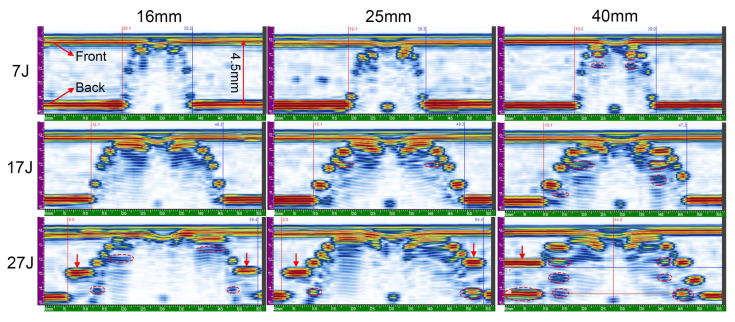
S-scan images from front surface.

Typical cross-section S-scans obtained with PAUT are presented in Figure 14, as recorded in the center of impact dent from front surface. In Figure 14, the distance from the front-surface echo to the back-surface echo on index axis reaches approximately 4.5 mm, complying with the real thickness. The dent damages can be seen at the middle of front-surface images, and near the impact point, damages seem to be more serious with compressive failure for high local impact stress. The size of internal damage increases with the rise in depth from front surface to back surface; therefor, a frustum-cone shape pattern can be identified more clearly than in the C-scans. The reference cursor (represented by red line in the left) and the measurement cursor (represented by blue in the right) were adopted to identify the maximum lateral size of the damaged area. The artefacts marked by red ellipses refer to repeated echoes caused by the first reflector, which can be detected by their regular pattern under a constant offset distance.

**Figure 15:**
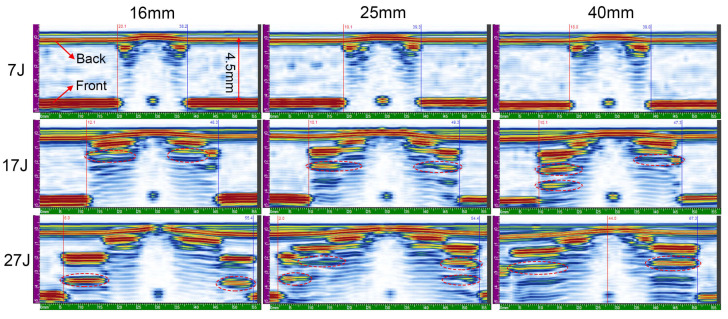
S-scan images from back surface.

For the phenomena of acoustic shadowing, the first delamination is difficult to look behind as everything behind is in the acoustic shadow. To get a more complete information of the internal damage, the S-scans were again recorded from the back surface and present a cross-section profile through the whole thickness of the plate (Figure 15). A bulge is identified at the back surface of all specimens directly just below the impact point, demonstrating that the damage occurs through the whole thickness. The maximum lateral size of the damage determined from the back-surface S-scans is identical to that obtained from front surface S-scans. The size of delamination near the back-surface is the same as the maximum lateral size at low impact energy, while the size is smaller at high energy with a sudden decrease.

**Figure 16:**
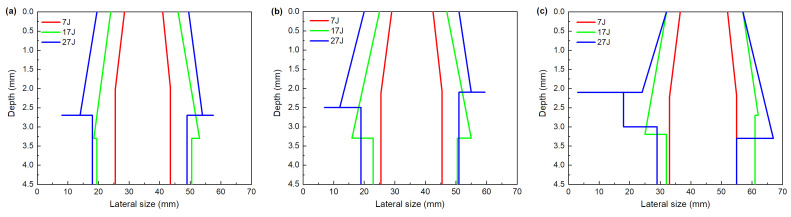
Cross-section damage outline with different impactor diameters: (**a**) 16 mm, (**b**) 25 mm, (**c**) 40 mm.

**Figure 17:**
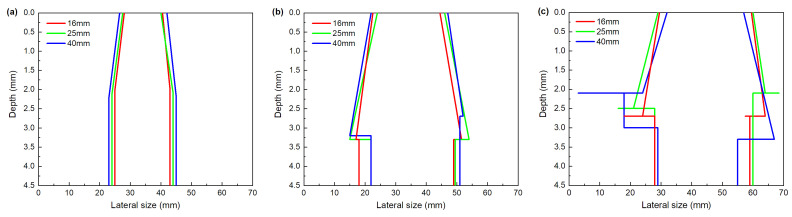
Cross-section damage outline with different impact energies: (**a**) 7 J, (**b**) 17 J, (**c**) 27 J.

Given the S-scans achieved from front and back surfaces, the cross-section damage outline through thickness can be obtained, as shown in Figure 16 and Figure 17, respectively representing different impactor diameters and impact energies. The cross-section outline and extension shape of the damage through the whole thickness can be observed here more intuitively. In Figure 16, for all impactor diameters, with the increase in impact energy, the lateral size increases. The cross-section outline exhibits different shapes under different impact energies. Under lower impact energy, the damage size in the plate is elevated with increasing depth, and then remains constant to the back surface. While at higher impact energy, after reaching the maximum in a certain depth, the damage size decreases abruptly.

It is suggested that the impactor diameter does not affect the cross-section shape (Figure 17). For all impact energies, the damage outlines are nearly the same with different impactor diameter, and damage size only has a little increase with the increase in the impactor diameter. The depth of maximum lateral size of internal damage presents some differences with different impact energy, which is located between 2 and 2.5 mm (Figure 17a), 3 and 3.5 mm (Figure 17b), 2 and 3 mm (Figure 17c), respectively. The value of maximum lateral size increases with the increase in the impact energy. Given the mentioned impactor displacement and absorbed energy, this finding may be attributed to the larger bending stress as impacted with large energy. The bending stress facilitates the extension of delamination at a direction parallel to the layer, as well as preventing the damage from propagating at the depth direction. It can be concluded that an energy threshold is identified, i.e., that when impacted energy is larger than it, the bending stress starts to significantly induce the internal damages, regardless of the impactor diameters.

## 4. Conclusions

In the present study, low velocity impact test was performed with different impactor diameters under different impact energies, to delve into its effect on the induced impact damage. Double-sided PAUT was applied to inspect reliable damage in the impacted CFRP laminates. Besides, impact response parameters and damage characteristics were determined to assess the impactor diameter effect on the low velocity impact as well as their correlations. According to the experimental studies, the following conclusions can be drawn:

1. The impactor diameters significantly affects the impact damage states in the low velocity impact event, both on the impact response parameters and damage characteristics. For the same impact energy, the absorbed energy and final displacement increases with the increase in impactor diameter.
2. The dent depth decreases with the increase of impactor diameters. For small impactor diameter, visual impact damage at the front surface is more serious than the large one under equienergetic impact, as well as the back surface.
3. For the lower impact energy (7 J and 17 J), the damage area are nearly the same with the three impact diameters, while at the higher energy level (27 J) it increases as the impactor diameter increases.
4. The dent depth and damage area has a negative relationship for the 27 J impact energy with different impactor diameters, while has no obvious correlation for lower impact energies.
5. The extension shape of the internal damage is slightly related to impactor diameter and only linked to the impact energy. With the increase in the impact energy, the damage shape varies from frustum-cone to pine-tree. For all impactor diameters, the critical delamination at large impact energy is underneath the middle of the laminate as impacted by the interaction between interlaminar stresses and bending stress.

## Figures and Tables

**Table 1:** Mechanical properties of T300/YH69.

E${}_{11T}$ (GPa)	E${}_{22T}$ (GPa)	G${}_{12}$ (GPa)	τ${}_{12}$ (MPa)	υ ${}_{12}$	σ${}_{11T}$ (MPa)	σ${}_{22T}$ (MPa)	σ${}_{11C}$ (MPa)	σ${}_{22C}$ (MPa)
140	9	4.6	70	0.32	1760	51	1100	130
